# Supplementary material for: Elecsys pTau217 plasma immunoassay detection of amyloid pathology in clinical cohorts
Source: Alzheimers Dement. 2026 Jan 15;22(1):e71009. doi: 10.1002/alz.71009 (PMC12805464; doi:10.1002/alz.71009)
Supplement: Supplementary file 2 — Supporting information [file ALZ-22-e71009-s003.docx]

**Supplementary Figure 1:** ROC analyses for plasma pTau217 measurements with respect to amyloid PET visual read status in (A) cognitively impaired and (B) cognitively unimpaired individuals.

| 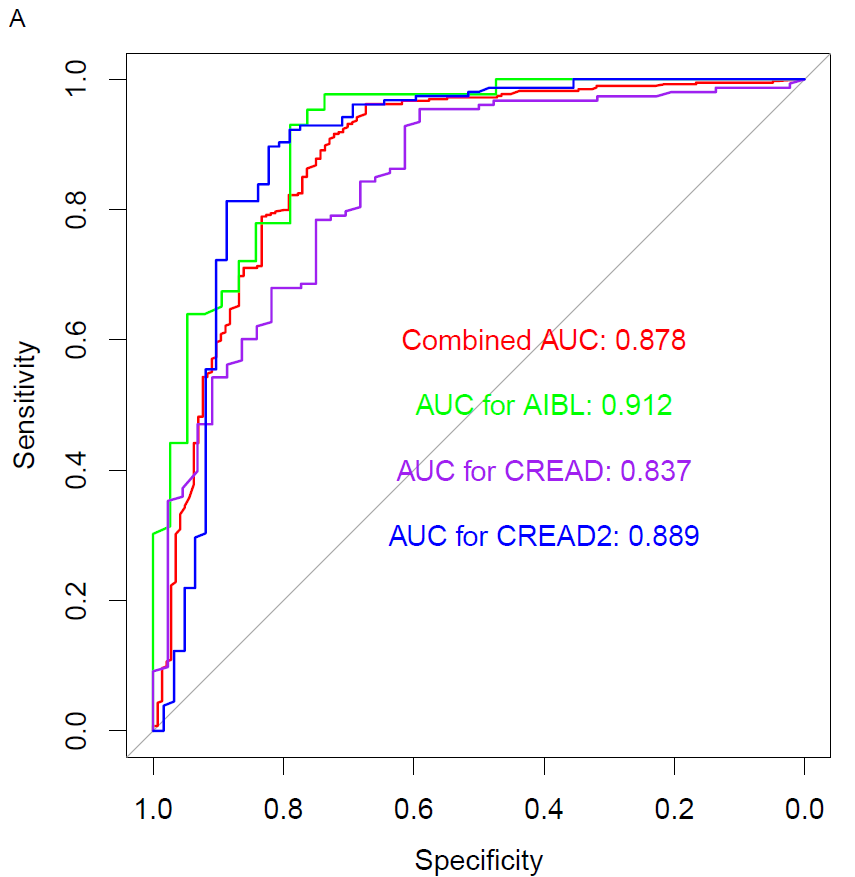 |
| --- |
| 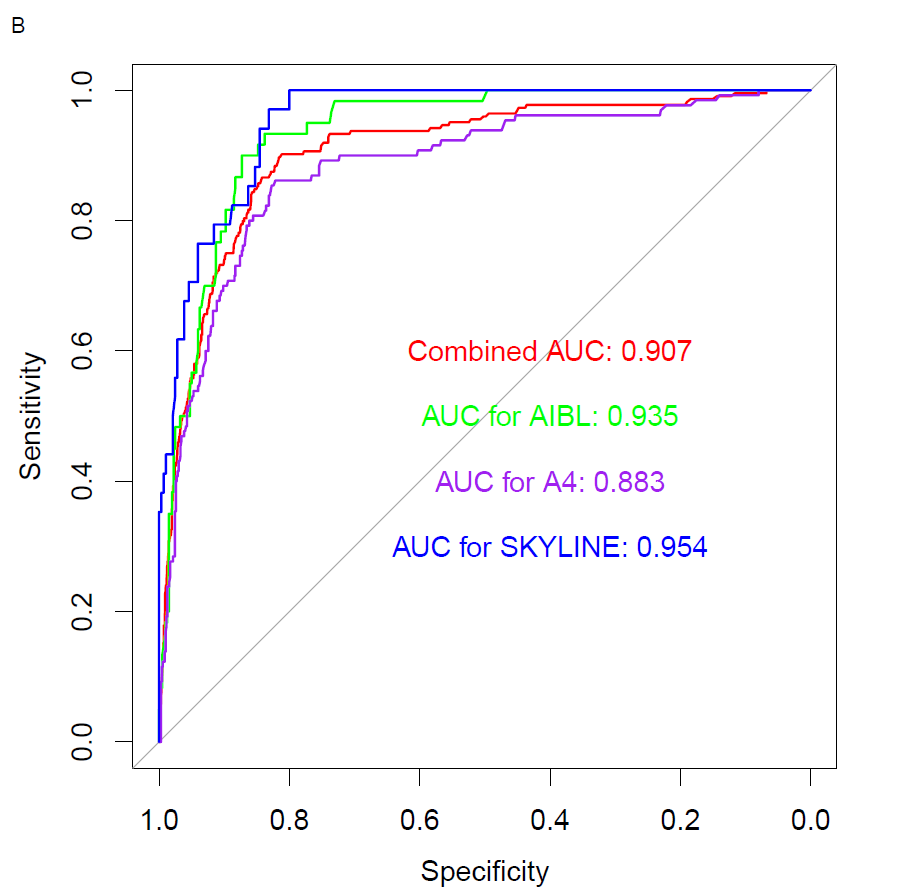 |

Abbreviations: A4, Anti-Amyloid Treatment in Asymptomatic Alzheimer’s Disease;
AIBL, Australian Imaging Biomarkers and Lifestyle; AUC, area under curve;
PET, positron emission tomography; pTau217, tau phosphorylated at threonine 217; ROC, receiver operating characteristic.

**Supplementary Figure 2:** Centiloid classifications vs amyloid PET visual read status
by diagnosis across the five clinical cohorts.

**
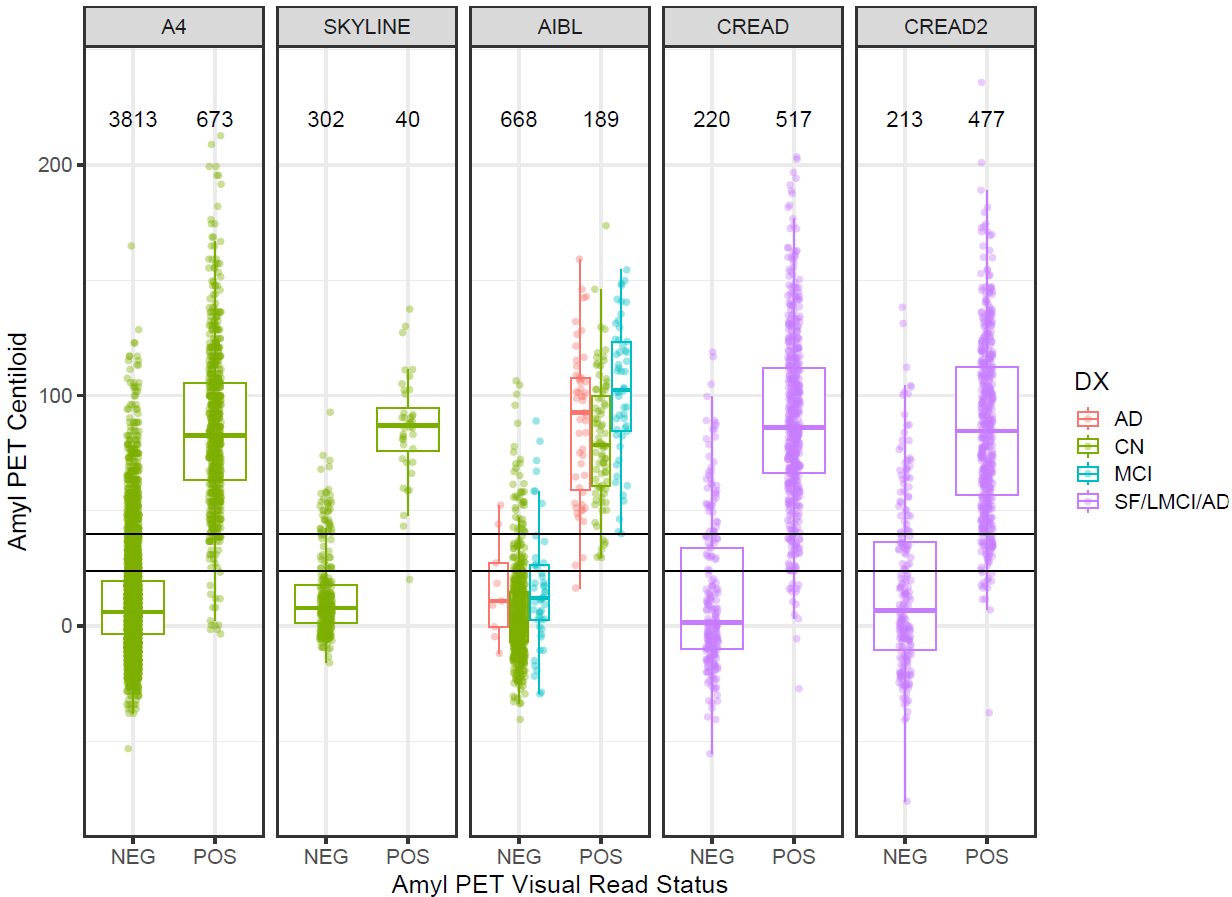
**

Horizontal black lines indicate centiloid cutoffs of ≥ 24 and ≥ 40.

Abbreviations: A4, Anti-Amyloid Treatment in Asymptomatic Alzheimer’s Disease;
AD, Alzheimer’s disease; AIBL, Australian Imaging Biomarkers and Lifestyle;
Amyl, amyloid; CN, cognitively normal; DX, diagnosis; LMCI, late mild cognitive impairment; MCI, mild cognitive impairment; NEG, negative; POS, positive;
PET, positron emission tomography; SF, screen fails.
